# Antimicrobial Activity of Lemongrass Essential Oil (*Cymbopogon flexuosus*) and Its Active Component Citral Against Dual-Species Biofilms of *Staphylococcus aureus* and *Candida* Species

**DOI:** 10.3389/fcimb.2020.603858

**Published:** 2020-12-22

**Authors:** Shanjun Gao, Guangzhi Liu, Jianguo Li, Jing Chen, Lina Li, Zhen Li, Xiulei Zhang, Shoumin Zhang, Rick Francis Thorne, Shuzhen Zhang

**Affiliations:** ^1^ Microbiome Laboratory, Henan Provincial People’s Hospital, People’s Hospital of Zhengzhou University, Zhengzhou, China; ^2^ Department of Dermatology, Henan Provincial People’s Hospital, People’s Hospital of Zhengzhou University, Zhengzhou, China; ^3^ Translational Research Institute of Henan Provincial People’s Hospital, Zhengzhou University, Zhengzhou, China; ^4^ Academy of Medical Sciences, Zhengzhou University, Zhengzhou, China; ^5^ School of Environmental & Life Sciences, University of Newcastle, Newcastle, NSW, Australia

**Keywords:** *Candida albicans*, *Candida tropicalis*, *Staphylococcus aureus*, dual-species biofilms, lemongrass essential oil, citral

## Abstract

Compared to mono-species biofilm, biofilms formed by cross-kingdom pathogens are more refractory to conventional antibiotics, thus complicating clinical treatment and causing significant morbidity. Lemongrass essential oil and its bioactive component citral were previously demonstrated to possess strong antimicrobial efficacy against pathogenic bacteria and fungi. However, their effects on polymicrobial biofilms remain to be determined. In this study, the efficacy of lemongrass (*Cymbopogon flexuosus*) essential oil and its bioactive part citral against dual-species biofilms formed by *Staphylococcus aureus* and *Candida* species was evaluated *in vitro*. Biofilm staining and viability test showed both lemongrass essential oil and citral were able to reduce biofilm biomass and cell viability of each species in the biofilm. Microscopic examinations showed these agents interfered with adhesive characteristics of each species and disrupted biofilm matrix through counteracting nucleic acids, proteins and carbohydrates in the biofilm. Moreover, transcriptional analyses indicated citral downregulated hyphal adhesins and virulent factors of *Candida albicans*, while also reducing expression of genes involved in quorum sensing, peptidoglycan and fatty acids biosynthesis of *S. aureus*. Taken together, our results demonstrate the potential of lemongrass essential oil and citral as promising agents against polymicrobial biofilms as well as the underlying mechanisms of their activity in this setting.

## Introduction

*Candida* species is one of the most prevalent fungal pathogens worldwide and prone to forming biofilms which have been extensively identified in human body, hospital environment, and medical materials (Cavalheiro and Teixeira, 2018). Infections caused by *Candida* biofilms usually exhibit high degree of tolerance to antifungal therapies and thus pose a serious threat to human health.

Although microorganisms are able to form single-species biofilm, it is more common that two or more microorganisms coexist in biofilms. Such multi-species biofilms can significantly increase the resistance of commensal microbes to conventional antimicrobic therapies and host immune system. For instance, ability of *S. aureus* to make biofilms is greatly enhanced when grown with *C. albicans*, and moreover, the susceptibility of *S. aureus* cells to routine antibiotics like vancomycin and oxacillin is markedly reduced (Nabb et al., 2019). Indeed, it is highly evident that infections caused by multi-species biofilms can lead to diseases with higher morbidity and mortality than those caused by single-species biofilm (Kong et al., 2016). For example, infections caused by dual-species biofilms of *C. albicans* and *S. aureus* have been frequently reported in the clinic, which makes the pathogens difficult to be eradicated (Qu et al., 2016). Therefore, exploration of effective agents targeting multi-species biofilms is urgently required.

Essential oils fractionally distilled from plants have drawn increased attention because of its multiple pharmacological properties like antibacterial, antifungal, and antiviral activities. Offering better biocompatibility and less side effects on human body, plant essential oils are regarded as potential alternatives to synthesis-based antibiotics and have been widely used in the treatment of cutaneous infections (Wińska et al., 2019). Citral, representing the most abundant component of lemongrass essential oil, is regarded as its biologically active constituent. Previous investigations have conformed lemongrass essential oil and citral possess strong activity against a broad spectrum of fungal and bacterial species (Naik et al., 2010; Shi et al., 2017). For instance, Silva et al. reported lemongrass essential oil and citral showed strong antifungal activity against several *Candida* species (Silva Cde et al., 2008). Furthermore, investigations performed by Zouhir et al. demonstrated lemongrass essential oil can eradicate the methicillin-resistant *Staphylococcus aureus* (MRSA) *in vitro* (Zouhir et al., 2016). Besides the studies on antimicrobial activities, one recent research investigated the in-depth molecular mechanism of citral against MRSA biofilm by using proteomic approach. This study unveiled citral inhibited the MRSA biofilm by differentially regulating the proteins involved in several biofilm related pathways (Valliammai et al., 2020).

Although several studies have confirmed the antimicrobial abilities of lemongrass essential oil and its major component citral, killing effects on multi-species biofilms as well as the working mechanism of action were not determined. In this study, we investigated the activity of lemongrass essential oil and citral against dual-species biofilms formed by *S. aureus* and *Candida* species. First, chemical constituents of lemongrass essential oil were analyzed. Secondly, effects of lemongrass essential oil and citral on biomass and cell viability of each species in polymicrobial biofilms were examined. Thirdly, effects of lemongrass essential oil and citral on the structure of biofilm matrix were assessed. Lastly, transcriptional responses of each species in dual-species biofilms were explored after citral treatment. These investigations not only shed new light on the therapeutic significance of lemongrass essential oil and citral but also uncovered the molecular impact of citral on dual-species biofilms.

## Materials and Methods

### Microbial Strains and Growth Cultures

*C. albicans* SC5314 (kindly donated by Professor Ruoyu Li from Peking university, China), *C. tropicalis* ATCC1369 and *S. aureus* ATCC25923 (both purchased from ATCC) were used in this research. YPD and BHI plus 1% glucose (OXOID, Basingstoke, England) medium were used for the growth of *C. albicans (*or *C. tropicalis)* and *S. aureus*, respectively. RPMI1640 medium (Thermo Fisher Scientific, USA) buffered to a pH of 7.0 with 0.165 M MOPS (Sangon Biotech, Shanghai, China) was used for growing the dual-species biofilms between *Candida* species and *S. aureus*.

### GC-MS Analysis

The chemical components of lemongrass (extracted from *C. flexuosus*) essential oil (doTERRA, USA) were analyzed by gas chromatography-mass spectra (GC-MS) system (Agilent gas chromatograph 6,890 coupled with Agilent 5973 mass selective detector, USA). The gas chromatographic separation was performed on a HP-INNOWAX capillary column (30 m × 0.25 mm i.d. with 0.25 μm film thickness, Agilent Technology, USA). The injector temperature was set at 250°C and high-purity helium was used as the carrier gas with flow rate of 1 ml/min. The sample was injected with a 1 μl volume and performed in a split mode (split ratio 30:1). The oven temperature was programmed as follows: initial temperature was maintained at 40°C for 2 min and then the temperature was increased to 250°C at 6°C/min and subsequently held for 10 min. The MS was performed in electron ionization mode (EI) with 70 eV ionization energy. The ion source temperature was set at 230°C with scan range from 30 to 300 m/z. The compounds were identified according to the NIST 2014 mass spectral library which is a standard mass spectrometry reference database released by National Institute of Standards and Technology (NIST) in 2014.

### Susceptibility Test of Planktonic Cells

Minimal inhibitory concentration (MIC) was performed by serial microdilutions based on previously described method with minor modifications (Miao et al., 2019; Niu et al., 2020). Specifically, *Candida* and staphylococcal cell suspensions were diluted to 1 × 10^5^ cells/ml in YPD and BHI respectively. Then aliquots of 100 µl of each species were separately transferred into each 96-well microtiter plates. Next, lemongrass essential oil was added in YPD or BHI to obtain final concentrations in the range of 0.0097–10 μg/ml; Similarly, citral was added in YPD or BHI to obtain final concentrations in the range of 0.0078–8 μg/ml. Following 24 h incubation at 37°C, the MIC value was determined as the lowest concentration (volume percent, v/v %) of lemongrass essential oil or citral which inhibited the visible growth of each species after overnight incubation. For each treatment condition, three replicates were performed and the experiment was repeated 3 times.

### Biomass and Viability Determination of Biofilms

All strains were stored in 10% glycerol at -80°C. *C. albicans* or *C. tropicalis* strain was inoculated on the Sabouraud’s Dextrose Agar (SDA) and *S. aureus* was grown on the Trypticase Soy Agar (TSA, OXOID, Basingstoke, England) overnight. Then single colony of *Candida* strains was inoculated in YPD and *S. aureus* was grown in BHI at 37°C overnight. The cultures were diluted to OD_600_ = 0.01 with RPMI1640, and seeded into 96-well microplates for 24 h at 37°C. The medium was refreshed with lemongrass essential oil or citral (mixture of *cis*-citral and *trans*-citral, MedChemExpress, USA) and co-incubated for another 24 h. Then, antimicrobial efficacy against dual-biofilms was evaluated in three aspects. Firstly, biofilm biomass reduction was quantified by the crystal violet (CV) staining. Briefly, dried biofilms were stained with 0.2% CV solution for 45 min, washed with water, and destained with 95% ethanol for 45 min. OD_570_ were measured in a microplate reader (BioTek, USA). The biomass reduction was calculated by the following formula. Reduction ratio (%) = (OD_untreated_-OD_treated_)/OD_untreated_ × 100%. Furthermore, reduction ratio of viable cells within biofilm was assessed by Cell Counting Kit-8 (CCK-8) method as previously described (Tan et al., 2019).The depth of color is directly proportional to cellular viability and OD_450_ was measured by microplate reader. The viability reduction ratio was calculated by the same formula as above. Besides, the reduction in viable counts CFU (Colony Forming Units) was detected by plate assay. For this, after treatment, biofilms were dissociated from the surface into PBS and then added onto YPD + vancomycin (2 mg/L) plates (select *C. albicans*) and TSA+ amphotericin B (2.5 mg/L) plates (select *S. aureus*) by serial-dilutions plating (Qu et al., 2016). The effectiveness to kill each species of biofilm was determined by calculating the direct reduction of viable cells [Reduction ratio % = (untreated CFU - treated CFU)/untreated CFU ×100%]. For each treatment condition, three replicates were performed and the experiment was repeated 3 times.

### Confocal Laser Scanning Microscopy

First, dual-species biofilms were grown on coverslips for 24 h, and supernatant was aspirated, and replaced with fresh RPMI-1640 containing lemongrass essential oil (0.0708% and 0.3125%, v/v) or citral (0.125% and 0.5%, v/v) for biofilm compositional analysis and lemongrass essential oil (0.3125%, v/v) or citral (0.5%, v/v) for viability analysis, and then grown for 24 h. Afterwards, biofilm components including carbohydrates, proteins and nucleic acids were visualized as previously described with modification (Shin and Eom, 2019). Briefly, the dual-species biofilms on coverslips were immersed with 4% (v/v) paraformaldehyde for 1 h and dried for 30 min. The amino groups of dual-species biofilm were stained 10 μg/ml fluorescein isothiocyanate isomer I (FITC) for 1 h. To visualize carbohydrates, 1 μg/ml of concanavalin A-Alex Fluor 594 conjugate (Con-A), which reacts with mannose and glucose of biofilm, was incubated for 30 min. Finally, nucleic acids were stained with 5 μg/ml of 4,6-diamidino-2-phenylindoldihydrochloride (DAPI) which binds to double-stranded DNA for 45 min. At the end of each staining, the biofilms were washed with PBS to remove dye residues. Besides, the biofilms were stained with the LIVE/DEAD BacLight Viability Kit (Thermo Fisher Scientific, USA) for 15 min according to the manufacturer’s protocol. Cellular fluorescence was evaluated with CLSM (Leica, SP8, Germany) and the image data was analyzed by Leica LAS AF Lite and ImageJ software (NIH, USA). For the image quantification, the fluorescence data were taken from four randomly selected fields with same size per sample and one of the four fields was chosen to represent the phenotype. In addition, the average of biofilm thickness was quantified by collecting the data from four z-stacks for each experiment condition. The CLSM experiment was independently repeated 3 times.

### Scanning Electron Microscopy

The *C. albicans/S. aureus* biofilms were first fixed with 2.5% glutaraldehyde and next dehydrated in 30%, 50%, 70%, 80%, 90%, and 95% dilution series of ethanol for 15 min, respectively. Next the samples were immersed in isoamyl acetate for 15 min and then attached to metallic stubs by carbon stickers and sputter-coated with gold. Finally, the morphology of prepared samples was observed by Hitachi SU8100 SEM microscopy (HITACHI, Japan). The representative SEM images were chosen from three biological replicates.

### qRT-PCR Analysis

1 ml overnight culture of bacteria and fungi were diluted to OD_600_ = 0.01, seeded into a 12-well polystyrene microplate, and grown for 24 h. After 24 h, the supernatant was refreshed with RPMI-1640 containing 0.5% citral, wells without treatment were set as control. After 24 h, fungal and bacterial mRNA were extracted according to Omega Yeast RNA kit and the Bacteria kit (Omega BioTek, USA) respectively. cDNA was obtained by FastKing RT Kit (Tiangen, China). The gene expression of *C. albicans* and *S. aureus*, were detected by SYBR Green quantitative Real-Time PCR assay (SuperReal PreMix Plus, Tiangen, China) and calculated using the formula 2^-ΔΔCt^. The reaction was run on ABI Step One qPCR system (Applied Biosystem, USA) as follows: initial step at 95°C for 15 min, followed by 40 cycles at 95°C for 10 s, 60°C for 30 s. Eighteen seconds rRNA was chosen as internal reference gene for fungi and 16s rRNA for bacteria to normalize the data respectively. The primers (**Table 1**) were synthesized by Shangya biotech company in China. qPCR analyses of each gene were performed in three biological replicates, each with three technical replicates.

**Table 1:** Primer sequences used in this study (5′-3′).

Gene name	Forward primer	Reverse primer
***Candida albicans***		
Internal control		
18s rRNA	AAACGGCTACCACATCCAAG	CCAAGCCCAAGGTTCAACTA
Hyphal adhesion		
*als3*	CAACTTGGGTTATTGAAACAAAAACA	AGAAACAGAAACCCAAGAACAACCT
*hwp1*	CATTGACTGAAAACACTCCAGG	GCAGGAATAGATGGTTGTGAAC
Virulence factor		
*sap1*	TTTCATCGCTCTTGCTATTGCTT	TGACATCAAAGTCTAAAGTGACAAAACC
*sap2*	TCCTGATGTTAATGTTGATTGTCAAG	TGGATCATATGTCCCCTTTTGTT
*sap3*	GGACCAGTAACATTTTTATGAGTTTTGAT	TGCTACTCCAACAACTTTCAACAAT
***Staphylococcus aureus***		
Internal control		
16s rRNA	CCATAAAGTTGTTCTCAGTT	CATGTCGATCTACGATTACT
Quorum sensing system		
*agrA*	GGAAATTGCCCTCGCAACTG	CCAACTGGGTCATGCTTACG
*hla*	GCGAAGTCTGGTGAAAACCC	CTTGGAACCCGGTATATGGCA
Fatty acid biosynthesis		
*acpP*	TGACCGTTTAGGTGTAGACGC	GCGATATCAAGTGAGTCAGCG
*accA*	TGCCACCTTCACCAATGACA	AAGCTGCTGAAGAACGTGGA
*fapR*	CATGTGCTGTTTGCTCAGGC	CGTGCTTCTGCTCTTACCGT
Peptidoglycan biosynthesis		
*murE*	GCCAAAGGTGCAACACATCA	TGTAAGCTGCTTCAAGGCCA
*pbp1*	AAGCAGCCTAAACGTGGTGA	GCATCCATGACAACCGCAAA

### Statistical Analysis

All experiments were performed at least 3 times with statistical significance of different treatment groups calculated by one-way analysis of variance (ANOVA, Bonferroni multiple-comparison test) or by students’ t test. Difference were considered significant if P value was less than 0.05. Statistical analyses and graphs were made using Microsoft Excel and GraphPad Prism 7.

## Results

### Chemical Compositions of Lemongrass Essential Oil

**Figure 1:**
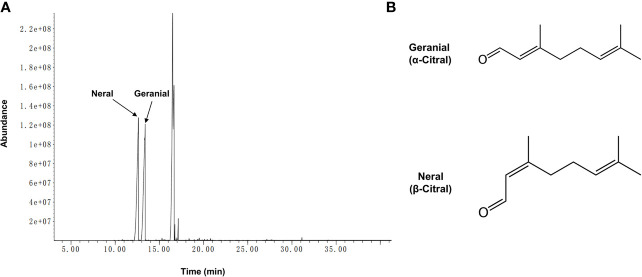
Chromatogram of lemongrass (*C. flexuosus*) essential oil and two geometric isomers of citral. **(A)** GC-MS profile of lemongrass essential oil. **(B)** Chemical formulas of geranial and neral which are two geometric isomers of citral.

The biologically active component of lemongrass essential oil is citral, which is mixture of two isomeric acyclic monoterpene aldehydes (neral and geranial). Compositional analysis by GC-MS identified a total number of 19 compounds with content exceeding 0.05%, making up ~99.6% of the lemongrass (*C. flexuosus*) essential oil (**Table 2**). According to the results, the primary components identified included ~29.4% geranial (*trans*-citral, α-citral) and ~30.4% neral (*cis*-citral, β-citral) (**Figure 1**). This is consistent with a previous study reporting citral as the dominant component of lemongrass essential oil, accounting for more than 60% of total compounds (Hadjilouka et al., 2017). Other identified high-content ingredients included caryophyllene (~25.4%) and indan-1,3-diol monoacetate (~7%). These results therefore validated the composition of lemongrass essential oil used in this study with citral confirmed as the most abundant compound.

**Table 2:** Major components of lemongrass essential oil (content>0.05%).

Retention time (min)	Component name	Percent of total (%)	Molecular formula	Molecular weight
*10.787*	*cis-Verbenol*	0.354	C_10_H_16_O	*152*
12.631	Neral (β-Citral)	30.395	C_10_H_16_O	152
12.654	Pulegone	0.072	C_10_H_16_O	152
12.761	Piperitone	0.161	C_10_H_16_O	152
13.39	Geranial (α-Citral)	29.364	C_10_H_16_O	152
14.629	Verbenol	0.373	C_10_H_16_O	152
15.299	Copaene	0.219	*C_15_*H*_24_*	*204*
15.536	Dihydromyrcene	0.403	C_10_H_18_	138
16.502	Caryophyllene	25.39	*C_15_*H*_24_*	*204*
16.645	Indan-1,3-diol monoacetate	7.021	C_11_H_14_O_4_	210
16.751	4,7-methano-1H-inden-5-ol,3a,4,5,6,7,7a-hexahydro-, acetate (Verdyl acetate)	1.283	C_12_H_16_O_2_	192
16.888	Dicyclopentenyl alcohol	0.466	C_10_H_14_O	150
17.137	Humulene	2.169	*C_15_*H*_24_*	*204*
18.358	δ -Cadinene	0.362	*C_15_*H*_24_*	*204*
19.301	Caryophyllenyl alcohol	0.235	C_15_H_26_O	222
19.508	Caryophyllene oxide	0.429	C_15_H_24_O	220
20.119	cis-Z-α -Bisabolene epoxide	0.241	C_15_H_24_O	220
20.783	α-Farnesene	0.437	*C_15_*H*_24_*	204
31.135	Eugenol	0.504	C_10_H_12_O_2_	164

### Susceptibility of Planktonic Cells to Lemongrass Essential Oil and Citral

To investigate the antimicrobial action of lemongrass essential oil and citral against planktonic phase of *Candida* species and *S. aureus*, the MIC values (v/v%) of each species and dual-species were determined (**Table 3**). The MIC of lemongrass essential oil against planktonic *C. albicans*, *C. tropicalis* and *S. aureus* were 0.0781%, 0.039%, and 0.0781%, respectively. The MIC of citral against *C. albicans*, *C. tropicalis*, and *S. aureus* were 0.0313%, 0.0156%, and 0.0313%, respectively. Regarding the dual-species planktonic *Candida* species and *S. aureus*, The MIC of lemongrass essential oil against *C. albicans*/*S. aureus* and *C. tropicalis*/*S. aureus* were 0.1563% and 0.0781%, respectively. The MIC of citral against *C. albicans*/*S. aureus* and *C. tropicalis*/*S. aureus* were 0.125% and 0.0313%, respectively. Therefore, both lemongrass essential oil and citral at low concentrations were enough to inhibit growth of planktonic *S. aureus* and *Candida* species.

**Table 3:** MIC (v/v %) of lemongrass essential oil (LEO) and citral against planktonic *S. aureus* and *Candida* species.

	LEO	Citral
*C. albicans*	0.0781	0.0313
*C. tropicalis*	0.039	0.0156
*S. aureus*	0.0781	0.0313
*C. albicans*/*S. aureus*	0.1563	0.125
*C. tropicalis*/*S. aureus*	0.0781	0.0313

### Antimicrobial Activity of Lemongrass Essential Oil and Citral Against Dual-Species Biofilms

**Figure 2:**
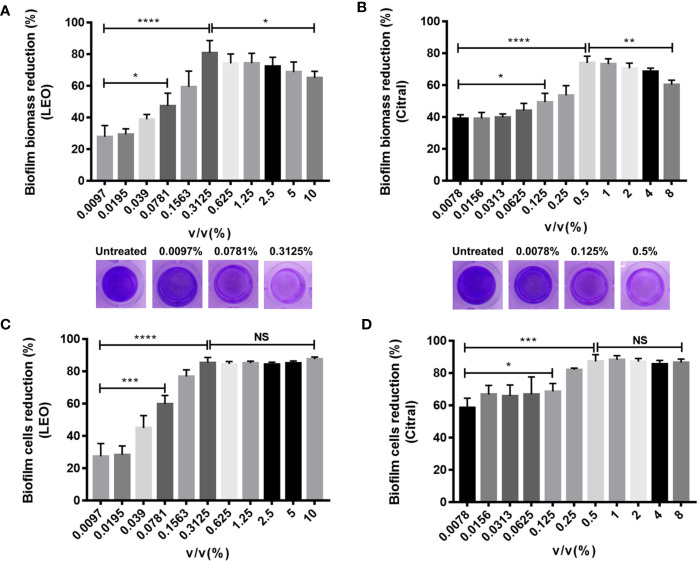
Antibiofilm activity of lemongrass essential oil or citral on dual-species biofilms of *C. albicans* and *S. aureus* after 24 h treatment. **(A)** Effect of lemongrass essential oil on biofilm biomass. The upper figure shows quantification data and the lower figures show representative biofilms stained by CV. **(B)** Effect of citral on biofilm biomass. The upper figure shows quantification data and the lower figures show the biofilms stained by crystal violet. **(C)** Effect of lemongrass essential oil on cell viability in biofilm. **(D)** Effect of citral on cell viability in biofilm. Data are shown as mean ± SD (n=3). *p < 0.05, **p < 0.01, ***p < 0.001, ****p < 0.0001, NS, No Significance; LEO, lemongrass essential oil.

To evaluate the efficacy of lemongrass essential oil and citral against dual-species biofilms formed by *Candida* species and *S. aureus*, crystal violet (CV) staining and CCK-8 test were performed to quantify biofilm biomass and viability respectively. The volume percent (v/v%) of lemongrass essential oil at the range from 0.097% to 10% were used to assess its antibiofilm activity. As present in **Figures 2A, C**, treatment by 0.3125% lemongrass essential oil significantly reduced the biofilm biomass by ~80% and cell viability by ~85%, showing the highest activity to eliminate biofilm. Interestingly, treatment with concentrations more than 0.3125% actually diminished the antibiofilm effects although the effects on cell viability remained maximal beyond 0.3125%. Similarly, experiments conducted with citral showed 0.5% treatment strongly reduced the biofilm biomass by ~74% but higher concentrations led to reduced activity (**Figure 2B**). At this concertation citral reduced cell viability by ~87%, but like the results with lemongrass essential oil, higher concentrations were similarly effective against biofilm viability (**Figure 2D**).

**Figure 3:**
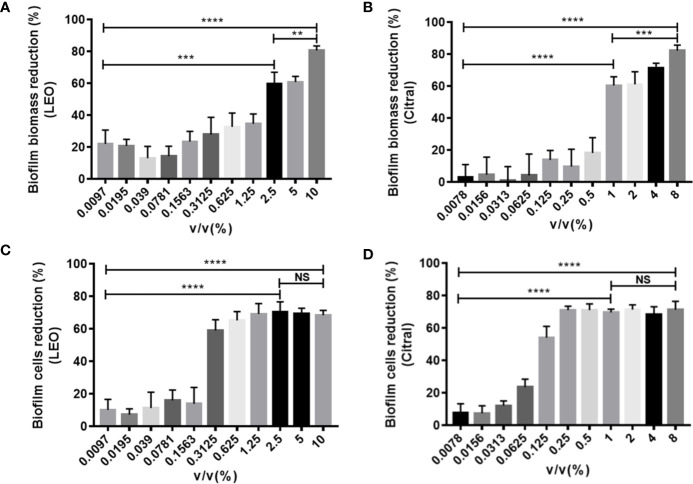
Antibiofilm activity of lemongrass essential oil or citral on dual-species biofilms of *C. tropicalis* and *S. aureus* after 24 h treatment. **(A)** Effect of lemongrass essential oil on biofilm biomass. **(B)** Effect of citral on biofilm biomass. **(C)** Effect of lemongrass essential oil on cell viability in biofilm. **(D)** Effect of citral on cell viability in biofilm. Data are shown as mean ± SD (n=3). **p < 0.01, ***p < 0.001, ****p < 0.0001, NS, No Significance; LEO, lemongrass essential oil.

The next series of experiments turned to examine the effects of the two agents on dual-species biofilms formed by *C. tropicalis* and *S. aureus.* Following the same approach, treatment with lemongrass essential oil significantly reduced the biofilm biomass, and in this instance the inhibition was greater with increasing concentrations with a maximal inhibition of ~80% at 10% (**Figure 3A**). Intriguingly, cell viability measurements showed again that 0.3125% was a threshold concentration for affecting dual-species biofilms of *C. tropicalis* and *S. aureus* (**Figure 3C**). Comparative assays with citral showed treatment with 8% citral maximally reduced biofilm biomass by ~82%, and with 0.25% or higher concentrations of citral evidently led to maximal cell viability reduction by ~71% (**Figures 3B, D** **)**.

### Antimicrobial Effects of Lemongrass Essential Oil and Citral on Residing Species in Dual-Species Biofilms

**Figure 4:**
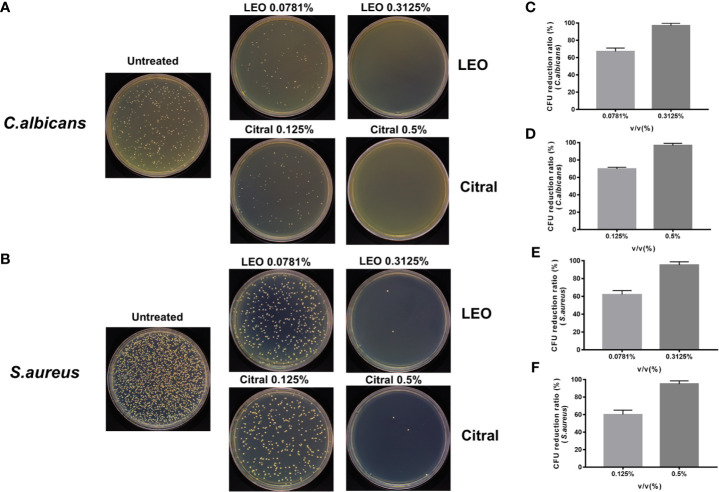
Antimicrobial effects of lemongrass essential oil or citral on the viability of residing species within dual-species biofilms. **(A)** Representative images of *Candida* CFU after 24 h treatment. **(B)** Representative images of staphylococcal CFU after 24 h treatment. **(C)** Comparison of the CFU treated by different concentrations of lemongrass essential oil and untreated CFU of *C. albicans*. **(D)** Comparison of the CFU treated by different concentrations of citral and untreated CFU of *C. albicans*. **(E)** Comparison of the CFU treated by different concentrations of LEO and untreated CFU of *S. aureus*. **(F)** Comparison of the CFU treated by different concentrations of citral and untreated CFU of *S. aureus*. LEO, lemongrass essential oil.

The preceding data provided evidence that lemongrass essential oil and citral could reduce both biofilm biomass and viability of *C. albicans*/*S. aureus* dual-species biofilms. However, these assays did not differentiate the effectiveness of these agents against the individual species inside the biofilm. To ascertain this, CFU measurements were undertaken after treating the dual-species biofilms with either lemongrass essential oil or citral (**Figures 4A, B**). Treating the biofilms with sub-optimal and optimal concentrations of lemongrass essential oil, 0.0781% and 0.3125%, respectively, resulted in killing rates of ~67% and ~97% for *C. albicans* in comparison to ~62% and ~95% for *S. aureus* (**Figures 4C, E**). Comparable experiments with citral at 0.125% killed ~70% *C. albicans* and ~60% *S. aureus*, while treatment with 0.5% citral killed ~96% *C. albicans* and ~94% *S. aureus* (**Figures 4D, F** **)**.

### Effects of Lemongrass Essential Oil and Citral on the Structure of Biofilm Matrix

**Figure 5:**
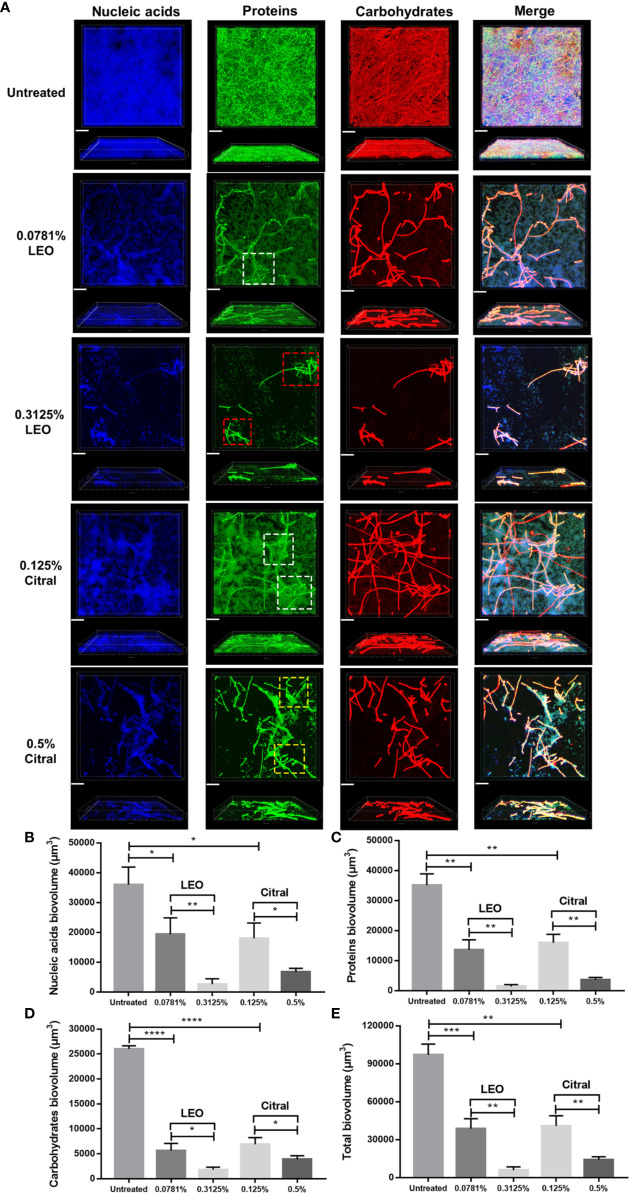
Compositional analysis of dual-species biofilm matrix after lemongrass essential oil or citral treatment. **(A)** Alterations of nucleic acids, proteins and carbohydrates in biofilm matrix were visualized by CLSM. The upper figures show top view and the lower figures show side view of biofilms; Three fluorescent dyes including DAPI, FITC and concanavalin A were applied to mark nucleic acids, proteins and carbohydrates, respectively. White, yellow and red squares (dotted lines) indicated high level adhesion, low level adhesion, and dissociation areas, respectively. The scale bar indicates 20 μm. **(B)** Comparison of the nucleic acids biovolume treated by LEO (or citral) and untreated control. **(C)** Comparison of the proteins biovolume treated by LEO (or citral) and untreated control. **(D)** Comparison of the carbohydrates biovolume treated by LEO (or citral) and untreated control. **(E)** Comparison of the total biovolume (nucleic acids + proteins + carbohydrates) treated by LEO (or citral) and untreated control. Data are shown as mean ± SD (n=4), *p < 0.05, **p < 0.01, ***p < 0.001, ****p < 0.0001. LEO, lemongrass essential oil.

To investigate whether lemongrass essential oil and citral impact the architecture of *C. albicans*/*S. aureus* dual-species biofilms, three major components of biofilm matrix, namely nucleic acids, proteins and carbohydrates, were examined by CLSM. As shown in **Figure 5A**, treatment of either lemongrass essential oil or citral resulted in obvious changes in the biofilm matrix. In particular, the dense network of filaments in untreated biofilms became more sparsely distributed after treatment. Treatment with optimal dose of lemongrass essential oil (0.3125%) exerted strong effect, substantially impairing the coadhesion between *C. albicans* and *S. aureus* compared to the sub-optimal treatment (white square versus red square in **Figure 5A**). However, compared to the sub-optimal treatment, application of optimal dose of citral (0.5%) also reduced the coadhesion while the effect was not significant as the lemongrass essential oil did (white square versus yellow square in **Figure 5A**). These observations were therefore largely consistent with the treatment outcomes, with lemongrass essential oil being marginally more effective than citral treatment, although both agents reduced matrix density.

On the basis of CLSM images, alteration of nucleic acid, protein and carbohydrate contents were quantified after treatment by these two agents. As shown in **Figure 5B**, compared to the sub-optimal treatment (0.0781% lemongrass essential oil and 0.125% citral), application of 0.3125% lemongrass essential oil and 0.5% citral significantly reduced the biovolume of nucleic acids by ~86% and ~63% respectively. Furthermore, these optimal treatments also caused sharp reductions in proteins by ~89% and ~77% (**Figure 5C**), as well as carbohydrates by ~68% and ~44% (**Figure 5D**), respectively. Overall, these treatments reduced the total biovolume of three main biofilm components by ~85% and ~65% (**Figure 5E**), respectively. Again, based on the measurable changes in biofilm architecture, these results implied that lemongrass essential oil showed slight advantages over citral in inhibiting the compositional biovolume of dual-species biofilms.

**Figure 6:**
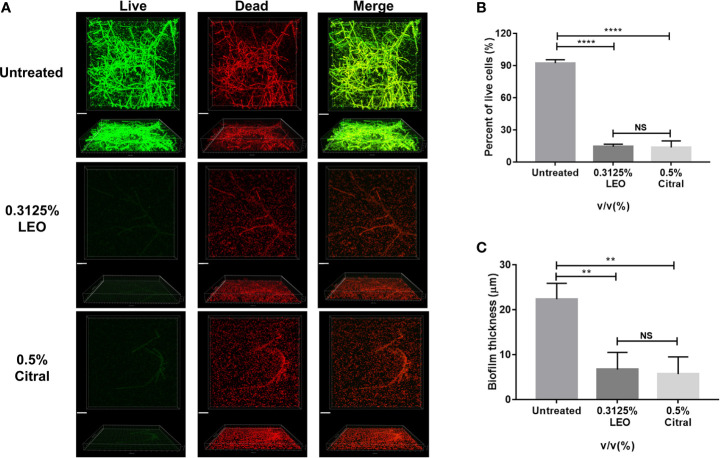
CLSM evaluation of live/dead ratio and biofilm thickness after 24 h exposure to lemongrass essential oil or citral. **(A)** Visualization of dual-species biofilms by live/dead cell staining. The upper figures show top view and the lower figures show side view of biofilms. **(B)** Quantification of the live cells in dual-species biofilms after lemongrass essential oil or citral treatment. **(C)** Quantification of the biofilm thickness after LEO or citral treatment. The scale bar indicates 20 μm; Data are shown as mean ± SD (n=4), **p < 0.01, ****p < 0.0001, NS, No Significance.

Next, live/dead cell staining was performed by CLSM to further investigate effects of lemongrass essential oil and citral on the biofilm structure. As shown in **Figure 6A**, exposure of biofilms to 0.3125% lemongrass essential oil and 0.5% citral resulted in striking reductions in viable cells present in biofilm. As with the biovolume measures, the CLSM images could also be analyzed to estimate the cell viability rates within the treated biofilms. Image analysis revealed that 0.3125% lemongrass essential oil and 0.5% citral reduced the percent of viable cells from ~ 92% to ~14% and ~13%, respectively (**Figure 6B**). Moreover, the values calculated by CLSM were fully consistent with the activities of lemongrass essential oil and citral measured in both CCK-8 and CFU assays (refer **Figures 2** and **4**). In addition, 0.3125% lemongrass essential oil and 0.5% citral treatments also dramatically reduced the biofilm thickness from ~22 μm to ~7 μm and ~6 μm, respectively (**Figure 6C**), consistent with the reduced content of three major matrix components after treatment.

### Effects of Lemongrass Essential Oil and Citral on Biofilm Ultrastructure

**Figure 7:**
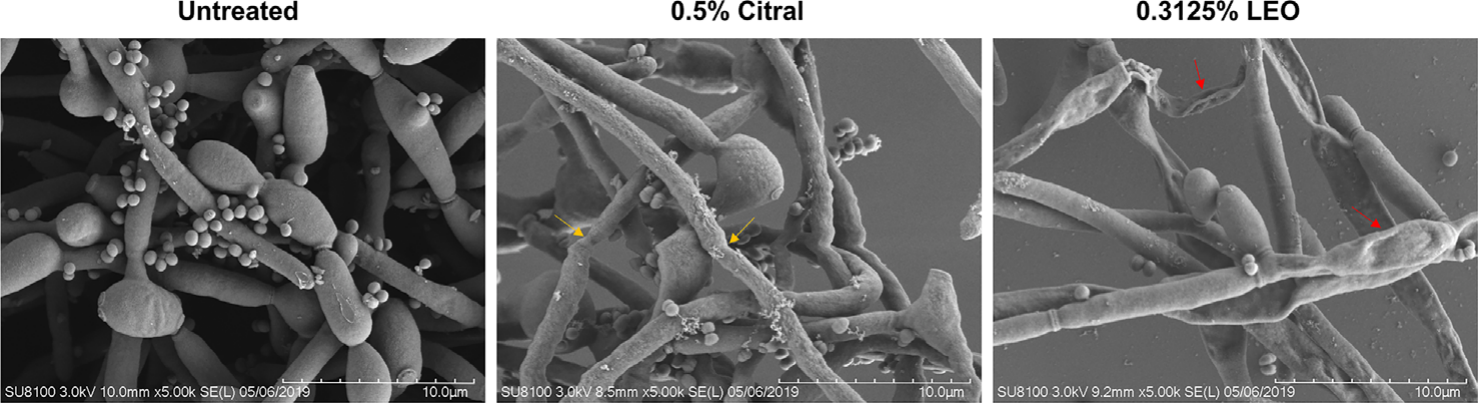
Observation of the inhibitory effects of lemongrass essential oil or citral on the microstructure of dual-species biofilms by SEM microscopy. LEO, lemongrass essential oil. Yellow arrows indicate fragile hyphae segmentation and red arrows indicate the shrunk surface on the *Candida* hyphae.

To visualize the alterations of micro-architecture of dual-species biofilms after exposure to lemongrass essential oil and citral, SEM microscopy was applied. As shown in **Figure 7**, following treatment of 0.5% citral, the number of adherent bacterial cells was obviously decreased. Additionally, compared with the hyphae without treatment, treated hyphae underwent clearly changes with thinner filaments, wrinkled surface and fragile hyphae segmentations (indicated by yellow arrows in the figure). Treatment by 0.3125% lemongrass essential oil resulted in even further reduction of adherent bacterial cells, as well as shrunk surface compared to that treated with 0.5% citral (indicated by red arrows in the figure). These results were in line with the CLSM observations shown in **Figure 5**, indicating both lemongrass essential oil and citral could impose inhibitory effects on the coadhesion between *C. albicans* and *S. aureus*, as well as changing the hyphal morphology of *C. albicans* in dual-species biofilms.

### Impacts of Citral on Gene Expressions in Dual-Species Biofilms

**Figure 8:**
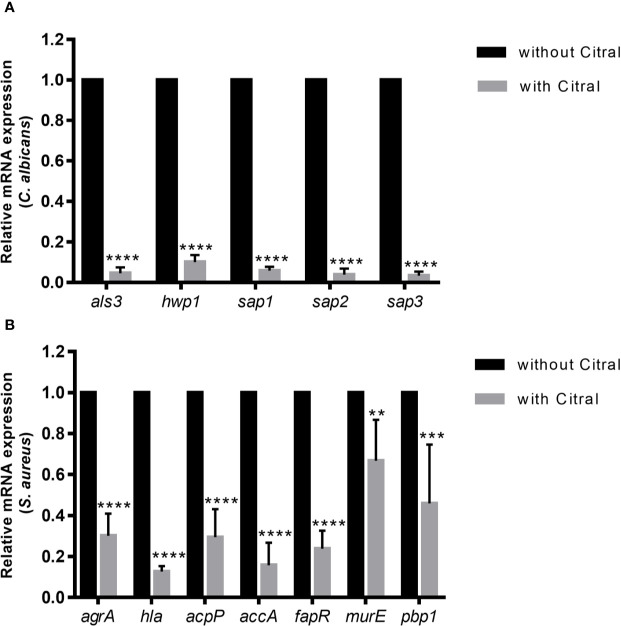
The expression profiles of related genes of each species in dual-species biofilms after 24 h exposure to 0.5% citral. **(A)** Expression levels of *als3*, *hwp1*, *sap1*, *sap2*, and *sap3* in *C. albicans*. **(B)** Expression levels of *agrA*, *hla*, *acpP*, *accA*, *fapR*, *murE*, and *pbp1* in *S. aureus*. The untreated biofilm was set as control.18s rRNA gene was used as internal control for *C. albicans* and 16s rRNA gene was used as internal control for *S. aureus*. Data are shown as mean ± SD (n=3), **p < 0.01, ***p < 0.001, ****p < 0.0001.

To dissect molecular responses of each species to citral, qPCR assay was performed to examine the transcriptional level of biofilm-related genes. The relative fold change in gene expressions of *C. albicans* and *S. aureus* were normalized to each housekeeping gene 18s rRNA and 16s rRNA respectively and calculated by the DeltaDeltaCt method. Comparing gene expression changes in dual-species biofilms between untreated and 0.125% citral treated samples showed remarkable downregulated expression of *Candida* adhesion related genes *als3* and *hwp1* by ~ 95% and ~ 90%, respectively (**Figure 8A**). Furthermore, *SAP* genes including *sap1*, *sap2*, and *sap3* which encode secreted aspartyl proteinases were significantly repressed by ~94.1%, ~96.2%, and ~96.8%, respectively. These data indicated citral counteracted *C. albicans* in dual-species biofilms through exerting effects on *Candida* adhesins and virulent factors.

As for the transcriptional levels of *S. aureus* in dual-species biofilms, treatment by citral critically influenced several biosynthetic pathways and signaling system of *S. aureus*. As shown in **Figure 8B**, treatment with 0.5% citral led to dramatically downregulation of central toxin regulator AgrA and α-toxin (encoded by *hla* gene) by ~ 69.8% and ~ 87.3%, respectively. Moreover, expression of *acpP*, *accA* and *fapR*, genes encoding crucial components involved in fatty acids biosynthesis, were also prominently repressed (~70.5%, ~84.3%, and ~76.2% reductions, respectively). In addition, expressions of MurE and Pbp1, which are essential for the peptidoglycan biosynthesis, were downregulated by ~33.2% and ~54%, respectively. Thus, these data indicated that citral inhibited *S. aureus* in dual-species biofilms by affecting quorum sensing system, fatty acids and peptidoglycan biosynthesis.

## Discussion

*S. aureus* and *C. albicans* are common opportunistic pathogens which cause serious systemic infections in humans. It is known that *S. aureus* and *C. albicans* can develop multi-species biofilms in order to increase the resistance of commensal cells to antibiotics treatment and host immune response (Nabb et al., 2019). Considering the life-threating infection imposed by multi-species biofilms and limited therapeutic options to treat such infection, it is urgent to search for novel and effective agents. Lemongrass essential oil and its major component citral have been reported to be able to inhibit the growth of a broad spectrum of pathogens including *S. aureus* and *Candida* species (Naik et al., 2010; Shi et al., 2017). Although both lemongrass essential oil and citral exhibit excellent antimicrobial effects, their effects on multi-species biofilms as well as antibiofilm mechanisms have not been revealed. In this study, we investigated the potential of lemongrass essential oil and citral against the dual-species biofilms formed by *S. aureus* and *Candida* species.

Susceptibility test of planktonic cells indicated low concentration of lemongrass essential oil (≥0.0781%) and citral (≥0.0313%) were sufficient to inhibit the planktonic growth of *C. albicans*, *C. tropicalis* and *S. aureus*. However, considering the relatively lower MIC compared to that of lemongrass essential oil, citral showed slightly advantage to inhibit the planktonic growth of the tested strains. Next, we examined the efficacy of lemongrass essential oil and citral against dual-species biofilms formed by *S. aureus* and *Candida* species. First, the test was performed on the dual-species biofilms of *S. aureus* and *C. albicans*. We found that treatment by 0.3125% lemongrass essential oil and 0.5% citral yielded the best performance, while higher concentration of both agents couldn’t improve the inhibitory effect, indicating a dose-optimal concentration for antibiofilm efficacy. However, although the same general properties of both lemongrass essential oil and citral were evident, the optimal concentration of 0.5% citral was found to be higher than the 0.3125% determined for the parental oil. The reasons for these are presently unclear but two possibilities maybe the effects of the citral isolation process or alternatively that other components of lemongrass essential oil also possess antibiofilm activity (Madeira et al., 2016). Regarding the *C. tropicalis*/*S. aureus* dual-species biofilms, the inhibitory effect of biofilm biomass was greater with increasing concentrations of these two agents, while 0.3125% lemongrass essential oil or 0.5% citral gave rise to the maximal killing effect on biofilm viability. Thus, collectively the data demonstrated that both lemongrass essential oil and citral could be used to eliminate dual-species biofilms to overall similar levels of efficiency. However, there are some differences according to the species composition of the fungal partners. The optimal reduction dosage for biofilms composed of *C. albicans*/*S. aureus* involves treatment with either 0.3125% lemongrass essential oil or 0.5% citral, respectively. This phenomenon is not evident for *C. tropicalis*/*S. aureus* biofilms since the responses were dose-dependent and the maximally effective dose of both lemongrass essential oil and citral were the highest used in the experimental range. Nevertheless, from the perspective of reducing biofilm viability, the effectively minimum doses of lemongrass essential oil and citral against either *C. tropicalis*/*S. aureus* or *C. albicans*/*S. aureus* dual-species biofilms were the same (0.3125% lemongrass essential oil and 0.5% citral, respectively).

Previous studies have reported that 0.125% lemongrass essential oil could inhibit biofilm formation of both methicillin susceptible *S. aureus* (MSSA) and methicillin resistant *S. aureus* (MRSA) strains *in vitro* (Adukwu et al., 2012). However, assuming that the effectiveness of preparations is similar, our data showed a higher concentration of lemongrass essential oil (0.3125%) was necessary to eliminate *C. albicans/S. aureus* dual-species biofilms, consistent with the idea that dual-species biofilms have increased resistance compared to mono-species biofilm. It is known that lemongrass essential oil is mainly composed of citral, which is a natural mixture of two geometric isomers and responsible for antimicrobial action (Somolinos et al., 2010; Verma et al., 2015). Although citral has been demonstrated to have strong antibacterial and antifungal activities (Lima et al., 2012; Shi et al., 2017), overdoses of citral can induce irritation and sensitization on human skin (Heydorn et al., 2003). For example, previous study found citral concentration higher than 1% could induce cytotoxicity in human fibroblast cells (Hayes and Markovic, 2002). Compared with citral, though lemongrass essential oil has less irritability to human body (Lalko and Api, 2006), the phototoxic and cytotoxic effects of lemongrass essential oil with high citral content (70%–90%) on the murine fibroblast cells and rabbit cornea derived cells have been reported (Dijoux et al., 2006). In addition, an early study on human subjects found the concentration of lemongrass essential oil above 4% could induce skin sensitization (Opdyke, 1976). By comparison, in our study, the optimal concentrations of both agents were much lower than that reported to be toxic, indicating the concentrations of both agents against dual-species biofilms were non-toxic to humans.

As discussed above, a higher concentration of 0.5% citral were required to achieve comparable efficacy to the optimal 0.3125% lemongrass essential oil against *C. albicans/S. aureus* dual-species biofilms, proposing that the unmodified formulation of lemongrass essential oil is the preferential form for topical applications.

Polymicrobial biofilm formation is attributed to synergistic effects of interspecies that enhance resistance of commensal microbes against antimicrobial agents. For example, in the context of dual-species biofilms, β-1,3-glucan, an important biofilm constituent produced by *C. albicans*, is able to protect MRSA from killing by vancomycin (Kong et al., 2016). To test whether dual-species biofilms provide protection to one or both residing species from the killing of lemongrass essential oil or citral, we assessed the number of viable cells for each species in biofilms. Our results demonstrated that lemongrass essential oil and citral displayed comparable effects against the growth of *C. albicans* and *S. aureus* in dual-species biofilms. These data suggested favorable properties of these agents since activity was evident against both species in the biofilm.

Different exopolymeric components secreted by *C. albicans* and *S. aureus* interact with each other to maintain the structural stability of biofilm matrix, giving rise to formation of three-dimensional network for shielding the microbial cells from environmental attack (Koo and Yamada, 2016; Lohse et al., 2018). The role of nucleic acids has been described as connecters to link different components in biofilm matrix and therefore indispensable for matrix integrity (Martins et al., 2010). Proteins, accounting for the most abundant component in biofilm matrix, are involved in various activities like amino acid metabolism, matrix homeostasis, and biofilm dispersal (Uppuluri et al., 2010; Zarnowski et al., 2014). Carbohydrates or exopolysaccharides, major components to constitute the scaffolds of biofilm matrix, are necessary for conferring the residing microbes tolerance against antibiotics (Pierce et al., 2017). Here, evaluation of lemongrass essential oil and citral showed their effectiveness not only relied on killing the commensal microbes, but also on disrupting the key components of three-dimensional (3D) structure, resulting in a thinner and less-dense biofilm.

Molecular responses of each species in the dual-species biofilms to citral were investigated by qPCR. The transcriptional levels of the critical genes involved in cell wall and membrane biosynthesis, quorum sensing signaling, biofilms formation and virulence factors were assessed after citral treatment. Citral was chosen over lemongrass essential oil for the molecular experiments given its relatively purified composition. For the transcriptional alterations of *C. albicans*, expression of hyphal adhesin Als3 and cell wall protein Hwp1, which were involved in the adherence of *S. aureus* to *C. albicans* in the biofilm matrix (Schlecht et al., 2015; Todd et al., 2019), were significantly downregulated by the citral treatment. In addition, expression of the secreted virulent factors such as aspartyl proteinases including SAP1, SAP2, and SAP3 were significantly reduced in response to citral treatment. These results were in agreement with the previous study showing treatment with Mentha × piperita essential oil reduced the expression of *SAP* family genes and adhesion gene *hwp1* in *C. albicans* (Benzaid et al., 2019). In *S. aureus*, accessory gene regulator (*agr*) system is the only quorum sensing system reported to mediate the interaction between *C. albicans* and *S. aureus* in dual-species biofilms (Todd et al., 2019). AgrA and α-toxin, two dispensable elements of *agr* system, have been proven to be responsible for the host lethality caused by *C. albicans/S. aureus* dual-species biofilms in a mouse infection model (Todd et al., 2019). Our results showed expression of *agrA* and the α-toxin encoding gene *hla* were significantly reduced by citral treatment, implying the *agr* system dependent toxin secretion was blocked. Additionally, we found transcriptional levels of several genes encoding important enzymes involved in fatty acids and peptidoglycan biosynthesis in *S. aureus* were remarkedly suppressed after citral treatment, consistent with previous finding that showed exposure of *Listeria monocytogenes* to lemongrass essential oil caused downregulation of the genes involved in fatty acid and peptidoglycan biosynthesis (Hadjilouka et al., 2017).

Although low concentration of lemongrass essential oil and citral have been corroborated to possess low toxicity to human skin, it is still necessary to perform *in vivo* experiment to test effects of these two agents in animal models. Thus, in our future work, we will evaluate the antimicrobial activity of these two agents against dual-species biofilms of *S. aureus* and *Candida* species in mouse model, as well as revealing the host immune response. Another limitation of current study is lack of global molecular expression profiling in response to the treatment, and therefore transcriptome analysis by RNA sequencing will be applied to decipher the molecular interactions between the species inside multi-species biofilms in our future study.

**Figure 9:**
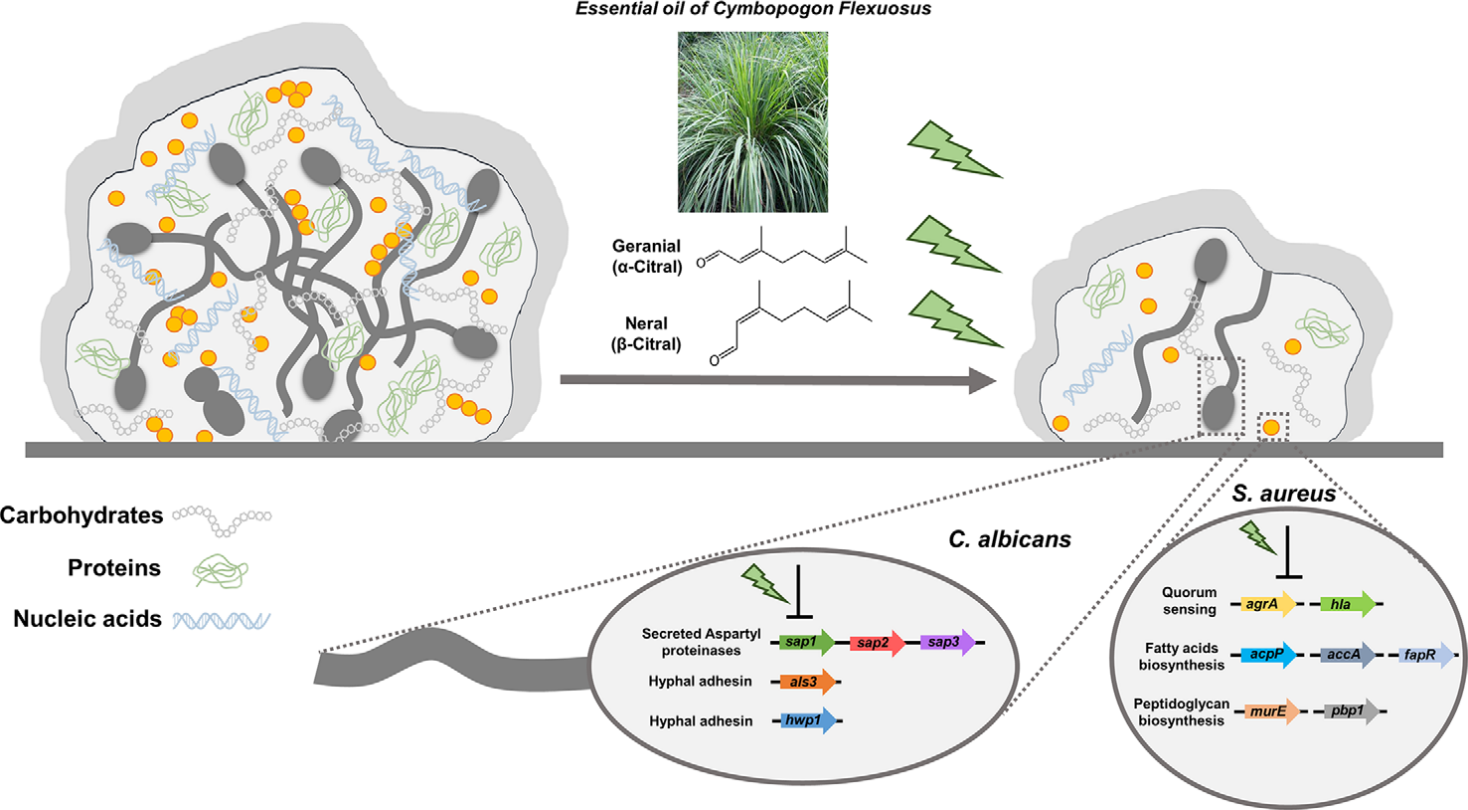
Schematic diagram of lemongrass essential oil or citral targeting the matrix composition and molecular systems of *C. albicans* and *S. aureus* in dual-species biofilms. Lemongrass essential oil and citral breaks the adhesion of staphylococcal cells to the *Candida* hyphae, as well as breaking the major constitutes of biofilm matrix. These two agents also repress the transcriptional levels of adhesion factors, virulent and biosynthetic pathways in *C. albicans* and *S. aureus* inside the dual-species biofilms.

In summary, we demonstrated lemongrass essential oil and citral were highly effective for eradicating the dual-species biofilms by hindering the interactions between *C. albicans* and *S. aureus* as well as breaking the matrix compositions of biofilms (**Figure 9**). Furthermore, it was found that treatment by citral suppressed hyphal adhesins and virulent factors in *C. albicans*, as well as the genes involved in quorum sensing, peptidoglycan, and fatty acids biosynthesis in *S. aureus*. Lastly, considering the low-cost and minimal risk to human, lemongrass essential oil and citral hold significant potential for pharmaceutical applications in the treatment of infections caused by polymicrobial biofilms.

## Data Availability Statement

The raw data supporting the conclusions of this article will be made available by the authors, without undue reservation.

## Author Contributions

Contributed to conceptualization and design: SG and SZZ. Contributed to the methodology and experiment execution: SG, GL, JL. Contributed to the GC-MS analysis: LL and ZL. Contributed to the microscopic visualization: XZ. Contributed to the image analysis: SMZ. Contributed to the qPCR experiment: JC. Contributed to the draft manuscript: SG and SZZ. Contributed to the manuscript revision: RT. SZZ was responsible for the supervision. All authors contributed to the article and approved the submitted version.

## Funding

This work was supported by “23456 Talents Project” from Henan Provincial People’s Hospital (Project No. ZC23456149).

## Conflict of Interest

The authors declare that the research was conducted in the absence of any commercial or financial relationships that could be construed as a potential conflict of interest.

## References

[B1] Adukwu E. C. Allen S. C. Phillips C. A. (2012). The anti-biofilm activity of lemongrass (*Cymbopogon flexuosus*) and grapefruit (*Citrus paradisi*) essential oils against five strains of *Staphylococcus aureus* . J. Appl. Microbiol. 113, 1217–1227. 10.1111/j.1365-2672.2012.05418.x 22862808

[B2] Benzaid C. Belmadani A. Djeribi R. Rouabhia M. (2019). The Effects of Mentha × piperita Essential Oil on *C. albicans* Growth, Transition, Biofilm Formation, and the Expression of Secreted Aspartyl Proteinases Genes. Antibiotics (Basel) 8, 10. 10.3390/antibiotics8010010 PMC6466576 30704020

[B3] Cavalheiro M. Teixeira M. C. (2018). Candida Biofilms: Threats, Challenges, and Promising Strategies. Front. Med. (Lausanne) 5, 28. 10.3389/fmed.2018.00028 29487851 PMC5816785

[B4] Dijoux N. Guingand Y. Bourgeois C. Durand S. Fromageot C. Combe C. (2006). Assessment of the phototoxic hazard of some essential oils using modified 3T3 neutral red uptake assay. Toxicol. Vitro 20, 480–489. 10.1016/j.tiv.2005.08.018 16219446

[B5] Hadjilouka A. Mavrogiannis G. Mallouchos A. Paramithiotis S. Mataragas M. Drosinos E. H. (2017). Effect of lemongrass essential oil on *Listeria monocytogenes* gene expression. LWT 77, 510–516. 10.1016/j.lwt.2016.11.080

[B6] Hayes A. J. Markovic B. (2002). Toxicity of Australian essential oil Backhousia citriodora (Lemon myrtle). Part 1. Antimicrobial activity and in vitro cytotoxicity. Food. Chem. Toxicol. 40, 535–543. 10.1016/s0278-6915(01)00103-x 11893412

[B7] Heydorn S. Menné T. Andersen K. E. Bruze M. Svedman C. White I. R. (2003). Citral a fragrance allergen and irritant. Contact Derm 49, 32–36. 10.1111/j.0105-1873.2003.00144.x 14641118

[B8] Kong E. F. Tsui C. Kucharíková S. Andes D. Van Dijck P. Jabra-Rizk M. A. (2016). Commensal Protection of *Staphylococcus aureus* against Antimicrobials by *Candida albicans* Biofilm Matrix. mBio 7, e01365–16. 10.1128/mBio.01365-16 27729510 PMC5061872

[B9] Koo H. Yamada K. M. (2016). Dynamic cell-matrix interactions modulate microbial biofilm and tissue 3D microenvironments. Curr. Opin. Cell. Biol. 42, 102–112. 10.1016/j.ceb.2016.05.005 27257751 PMC5064909

[B10] Lalko J. Api A. M. (2006). Investigation of the dermal sensitization potential of various essential oils in the local lymph node assay. Food. Chem. Toxicol. 44, 739–746. 10.1016/j.fct.2005.10.006 16324777

[B11] Lima I. O. De Medeiros Nóbrega F. De Oliveira W. A. De Oliveira Lima E. Albuquerque Menezes E. Afrânio Cunha F. (2012). Anti-*Candida albicans* effectiveness of citral and investigation of mode of action. Pharm. Biol. 50, 1536–1541. 10.3109/13880209.2012.694893 23116193

[B12] Lohse M. B. Gulati M. Johnson A. D. Nobile C. J. (2018). Development and regulation of single- and multi-species *Candida albicans* biofilms. Nat. Rev. Microbiol. 16, 19–31. 10.1038/nrmicro.2017.107 29062072 PMC5726514

[B13] Madeira P. L. Carvalho L. T. Paschoal M. A. De Sousa E. M. Moffa E. B. Da Silva M. A. (2016). In vitro Effects of Lemongrass Extract on *Candida albicans* Biofilms, Human Cells Viability, and Denture Surface. Front. Cell. Infect. Microbiol. 6, 71. 10.3389/fcimb.2016.00071 27446818 PMC4923188

[B14] Martins M. Uppuluri P. Thomas D. P. Cleary I. A. Henriques M. Lopez-Ribot J. L. (2010). Presence of extracellular DNA in the *Candida albicans* biofilm matrix and its contribution to biofilms. Mycopathologia 169, 323–331. 10.1007/s11046-009-9264-y 20012895 PMC3973130

[B15] Miao X. Liu H. Zheng Y. Guo D. Shi C. Xu Y. (2019). Inhibitory Effect of Thymoquinone on *Listeria monocytogenes* ATCC 19115 Biofilm Formation and Virulence Attributes Critical for Human Infection. Front. Cell. Infect. Microbiol. 9, 304. 10.3389/fcimb.2019.00304 31508379 PMC6718631

[B16] Nabb D. L. Song S. Kluthe K. E. Daubert T. A. Luedtke B. E. Nuxoll A. S. (2019). Polymicrobial Interactions Induce Multidrug Tolerance in *Staphylococcus aureus* Through Energy Depletion. Front. Microbiol. 10, 2803. 10.3389/fmicb.2019.02803 31866973 PMC6906149

[B17] Naik M. I. Fomda B. A. Jaykumar E. Bhat J. A. (2010). Antibacterial activity of lemongrass (*Cymbopogon citratus*) oil against some selected pathogenic bacterias. Asian Pac J. Trop. Med. 3, 535–538. 10.1016/S1995-7645(10)60129-0

[B18] Niu C. Wang C. Yang Y. Chen R. Zhang J. Chen H. (2020). Carvacrol Induces *Candida albicans* Apoptosis Associated With Ca(2+)/Calcineurin Pathway. Front. Cell. Infect. Microbiol. 10, 192. 10.3389/fcimb.2020.00192 32426298 PMC7203418

[B19] Opdyke D. L. (1976). Inhibition of sensitization reactions induced by certain aldehydes. Food. Cosmet Toxicol. 14, 197–198. 10.1016/s0015-6264(76)80424-5 950213

[B20] Pierce C. G. Vila T. Romo J. A. Montelongo-Jauregui D. Wall G. Ramasubramanian A. (2017). The *Candida albicans* Biofilm Matrix: Composition, Structure and Function. J. Fungi (Basel) 3, 14. 10.3390/jof3010014 28516088 PMC5431293

[B21] Qu Y. Locock K. Verma-Gaur J. Hay I. D. Meagher L. Traven A. (2016). Searching for new strategies against polymicrobial biofilm infections: guanylated polymethacrylates kill mixed fungal/bacterial biofilms. J. Antimicrob Chemother 71, 413–421. 10.1093/jac/dkv334 26490013

[B22] Schlecht L. M. Peters B. M. Krom B. P. Freiberg J. A. Hansch G. M. Filler S. G. (2015). Systemic Staphylococcus aureus infection mediated by *Candida albicans* hyphal invasion of mucosal tissue. Microbiology 161, 168–181. 10.1099/mic.0.083485-0 25332378 PMC4274785

[B23] Shi C. Sun Y. Liu Z. Guo D. Sun H. Sun Z. (2017). Inhibition of *Cronobacter sakazakii* Virulence Factors by Citral. Sci. Rep. 7, 43243. 10.1038/srep43243 28233814 PMC5324112

[B24] Shin D. S. Eom Y. B. (2019). Efficacy of zerumbone against dual-species biofilms of *Candida albicans* and *Staphylococcus aureus* . Microb. Pathog. 137, 103768. 10.1016/j.micpath.2019.103768 31585154

[B25] Silva Cde B. Guterres S. S. Weisheimer V. Schapoval E. E. (2008). Antifungal activity of the lemongrass oil and citral against *Candida* spp. Braz. J. Infect. Dis. 12, 63–66. 10.1590/s1413-86702008000100014 18553017

[B26] Somolinos M. García D. Condón S. Mackey B. Pagán R. (2010). Inactivation of *Escherichia coli* by citral. J. Appl. Microbiol. 108, 1928–1939. 10.1111/j.1365-2672.2009.04597.x 19891710

[B27] Tan Y. Leonhard M. Moser D. Ma S. Schneider-Stickler B. (2019). Antibiofilm efficacy of curcumin in combination with 2-aminobenzimidazole against single- and mixed-species biofilms of *Candida albicans* and *Staphylococcus aureus* . Colloids Surf B. Biointerfaces 174, 28–34. 10.1016/j.colsurfb.2018.10.079 30412864

[B28] Todd O. A. Fidel P. L. Jr. Harro J. M. Hilliard J. J. Tkaczyk C. Sellman B. R. (2019). *Candida albicans* Augments *Staphylococcus aureus* Virulence by Engaging the Staphylococcal agr Quorum Sensing System. MBio 10, e00910–19. 10.1128/mBio.00910-19 31164467 PMC6550526

[B29] Uppuluri P. Chaturvedi A. K. Srinivasan A. Banerjee M. Ramasubramaniam A. K. Kohler J. R. (2010). Dispersion as an important step in the *Candida albicans* biofilm developmental cycle. PLoS Pathog. 6, e1000828. 10.1371/journal.ppat.1000828 20360962 PMC2847914

[B30] Valliammai A. Sethupathy S. Ananthi S. Priya A. Selvaraj A. Nivetha V. (2020). Proteomic profiling unveils citral modulating expression of IsaA, CodY and SaeS to inhibit biofilm and virulence in methicillin-resistant *Staphylococcus aureus* . Int. J. Biol. Macromol. 158, 208–221. 10.1016/j.ijbiomac.2020.04.231 32360467

[B31] Verma R. K. Verma R. S. Chauhan A. Bisht A. (2015). Evaluation of essential oil yield and chemical composition of eight lemongrass (*Cymbopogon* spp.) cultivars under Himalayan region. J. Essent Oil Res. 27, 197–203. 10.1080/10412905.2015.1014936

[B32] Wińska K. Mączka W. Łyczko J. Grabarczyk M. Czubaszek A. Szumny A. (2019). Essential Oils as Antimicrobial Agents-Myth or Real Alternative? Molecules 24, 2130. 10.3390/molecules24112130 PMC6612361 31195752

[B33] Zarnowski R. Westler W. M. Lacmbouh G. A. Marita J. M. Bothe J. R. Bernhardt J. (2014). Novel entries in a fungal biofilm matrix encyclopedia. MBio 5, e01333–e01314. 10.1128/mBio.01333-14 25096878 PMC4128356

[B34] Zouhir A. Jridi T. Nefzi A. Ben Hamida J. Sebei K. (2016). Inhibition of methicillin-resistant *Staphylococcus aureus* (MRSA) by antimicrobial peptides (AMPs) and plant essential oils. Pharm. Biol. 54, 3136–3150. 10.1080/13880209.2016.1190763 27246787

